# A fully human chimeric antigen receptor with potent activity against cancer cells but reduced risk for off-tumor toxicity

**DOI:** 10.18632/oncotarget.4071

**Published:** 2015-06-19

**Authors:** De-Gang Song, Qunrui Ye, Mathilde Poussin, Lin Liu, Mariangela Figini, Daniel J. Powell

**Affiliations:** ^1^ Ovarian Cancer Research Center, Department of Obstetrics and Gynecology, Perelman School of Medicine, University of Pennsylvania, Philadelphia, PA, USA; ^2^ Department of Experimental Oncology and Molecular Medicine, Fondazione IRCCS Istituto Nazionale dei Tumori, Milan, Italy; ^3^ Department of Pathology and Laboratory Medicine, Abramson Cancer Center, Perelman School of Medicine, University of Pennsylvania, Philadelphia, PA, USA

**Keywords:** folate receptor alpha, chimeric antigen receptor, adoptive immunotherapy, ovarian cancer, T cells

## Abstract

Chimeric antigen receptors (CARs) can redirect T cells against antigen-expressing tumors in an HLA-independent manner. To date, various CARs have been constructed using mouse single chain antibody variable fragments (scFvs) of high affinity that are immunogenic in humans and have the potential to mediate “on-target” toxicity. Here, we developed and evaluated a fully human CAR comprised of the human C4 folate receptor-alpha (αFR)-specific scFv coupled to intracellular T cell signaling domains. Human T cells transduced to express the C4 CAR specifically secreted proinflammatory cytokine and exerted cytolytic functions when cultured with αFR-expressing tumors *in vitro*. Adoptive transfer of C4 CAR T cells mediated the regression of large, established human ovarian cancer in a xenogeneic mouse model. Relative to a murine MOv19 scFv-based αFR CAR, C4 CAR T cells mediated comparable cytotoxic tumor activity *in vitro* and *in vivo* but had lower affinity for αFR protein and exhibited reduced recognition of normal cells expressing low levels of αFR. Thus, T cells expressing a fully human CAR of intermediate affinity can efficiently kill antigen-expressing tumors *in vitro* and *in vivo* and may overcome issues of transgene immunogenicity and “on-target off-tumor” toxicity that plague trials utilizing CARs containing mouse-derived, high affinity scFvs.

## INTRODUCTION

The adoptive transfer of T cells expressing chimeric antigen receptors (CARs), or “T bodies”, has emerged as a powerful approach for cancer therapy [1]. CARs are comprised of an antigen-specific single-chain antibody variable fragment (scFv) fused to intracellular signaling domains derived from receptors involved in lymphocyte activation [2, 3]. CARs can functionally redirect T cells with high specificity to various surface antigens on tumor cells independent of MHC restriction and antigen processing, and therefore bypass major mechanisms by which tumors escape immune recognition [3]. The effectiveness of CAR therapy is evidenced by the complete eradication of CD19-expressing hematological malignancies following the adoptive transfer of autologous T cells engineered to express a CD19 redirected CAR that is observed in multiple independent trials [4–8].

Folate receptor α (αFR) is an attractive antigen for CAR-T cell therapy because αFR expression is over expressed in approximately 90% of ovarian carcinomas [9, 10], as well as in cancers of the endometrium [10], kidney [10], breast [11], lung [12], pancreas [10], colorectal cancer [13] and mesothelioma [14], and its expression is not affected by prior administration of chemotherapy [15]. In normal organs, such as kidney, lung, breast, and salivary glands, αFR expression is null or low and restricted to the apical surface of polarized epithelial cells [16], where it appears to be inaccessible to circulating anti-αFR antibodies and folic acid conjugates. CAR-T cell therapy was first tested in ovarian cancer [17], where administration of T cells engineered to express an anti-αFR CAR composed of the murine MOv18 scFv and a CD3z endodomain was shown to be feasible but did not induce tumor regression due to the poor persistence of the gene-modified T cells. Moreover, a serum inhibitory factor, likely human anti-mouse antibodies (HAMA), was detected in the serum of patients following treatment and shown to dampen CAR T cell function [17]. Elsewhere, HAMA to murine scFv on CAR T cells has resulted in severe anaphylactic response under a delayed dosing regimen [18], further exemplifying the need for construction and testing of human scFv based CARs. We have recently demonstrated that incorporation of costimulatory signaling domains, such as CD137 (4-1BB), CD28 or CD27, into a αFR-specific CAR overcomes the limitations of past CAR approaches by improving the persistence and anti-tumor activity of transferred CAR T cells *in vivo* [19, 20]. However, the anti-αFR scFv used in these studies was derived from the high affinity murine anti-human monoclonal antibody MOv19 and therefore runs the risk of being immunogenic in humans, and dampening the persistence and activity of αFR CAR T cells *in vivo*. The successful conversion of the murine MOv19 antibody to a fully human antibody, referred to as C4, through guided selection and chain shuffling techniques [21] makes the construction of a fully human anti-αFR CAR possible.

In this study, we constructed a novel fully human anti-αFR C4 CAR to reduce the risk of potential CAR transgene immunogenicity. The relative affinities of C4 and MOv19 in soluble scFv format has not been reported, however the binding affinity of the human C4 Fab fragment (2 × 10^7^ M^−1^) is approximately five-fold less than that of the high affinity murine MOv19 antibody. Even so, C4 retains its specificity for αFR and its K(d) of < 10^8^ M^−1^ is predicted to confer exclusive activation of CAR upon encounter with tumor cells bearing elevated amounts of surface αFR. The targeting domain is linked to a combined intracellular CD27 and CD3z signaling chain to further enhance the efficacy of this receptor (referred to hereafter as “C4-27z”). Here, we show that primary human T cells bearing the fully human C4-27z CAR specifically react against αFR^pos^ tumor cells *in vitro*, and mediate the regression of established human αFR^pos^ tumor in xenogeneic mouse models of advanced subcutaneous or intraperitoneal ovarian cancer. In addition, T cells expressing the C4-27z CAR possess antitumor activity *in vitro* and *in vivo* that is similar to that achieved using T cells expressing the murine MOv19-27z CAR. Importantly, the C4-27z CAR has reduced activity against normal cells bearing low level antigen and may decrease the potential risk of on-antigen, off-tumor toxicity. These results provide the rationale for the clinical investigation of fully human C4 CAR T cell therapy for the safe and effective treatment of a wide spectrum of αFR-expressing malignancies.

## RESULTS

### Construction and expression of fully human C4 CAR

**Figure 1:**
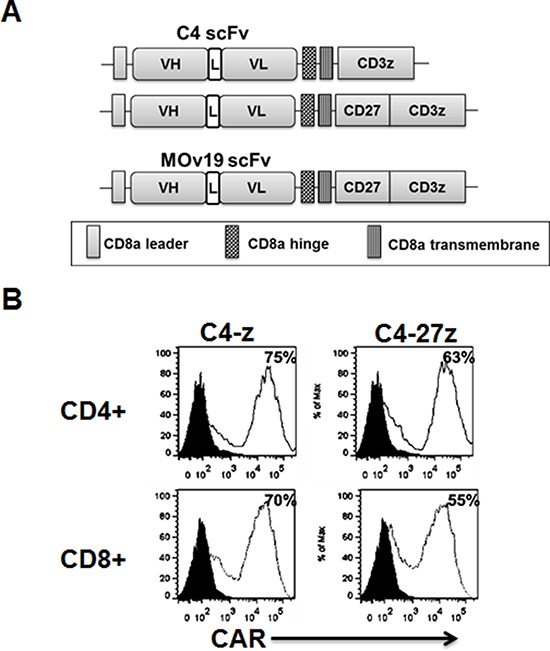
Generation of folate receptor alpha (αFR)-specific fully human chimeric antigen receptor (CAR) T cells **A.** Schematic representation of C4 based CAR constructs containing the CD3ζ cytosolic domain alone (C4-z) or in combination with the CD27 costimulatory module (C4-27z). The murine anti-human αFR MOv19-27z CAR is also shown. **B.** Transduced T cells consisted of CD4- and CD8-positive cells with both subsets expressing C4 CARs.C4 CAR expression (open histograms) was detected via biotin-labeled rabbit anti-human IgG (H+L) staining followed by streptavidin-phycoerythrin after transduction with lentivirus compared to untransduced (UNT) T cells (filled blackhistograms). Transduction efficiencies are indicated with the percentage of CAR expression in parentheses. ScFv, single-chain antibody variable fragment; L, linker; C4, anti-αFR scFv; VH, variable H chain; VL, variable L chain; TM, transmembrane region.

The fully human anti-human αFR-specific C4 Fab (referred to as C4) was previously described [21]. C4 CAR constructs comprised of a C4 scFv linked to a CD8α hinge and transmembrane region, followed by a CD3ζ signaling moiety alone (C4-z) or in tandem with the CD27 intracellular signaling motif were generated (C4-27z; Figure 1A) using CAR backbones described previously [19]. A previously described anti-CD19 CAR containing CD27 with CD3ζ signaling motifs in tandem (CD19-27z) was used as an antigen-specificity control [19, 22]. Primary human CD4+ or CD8+ T cells were efficiently transduced with recombinant lentiviral vectors to express C4 CAR with transduction efficiencies of about 50–80% (Figure 1B), and equilibrated to similar transduction efficiencies by adding untransduced(UNT) T cells for all functional assays.

### C4 CAR T cells specifically recognize αFR^pos^ ovarian cancer cells

**Figure 2:**
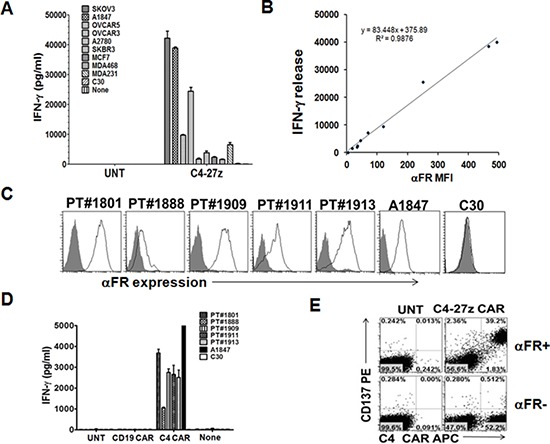
C4 CAR T cells secrete Th1 proinflammatory cytokine in response to tumor cell line and primary ovarian cancer cell surface associated αFR **A.** C4-27z CAR but not UNT T cells (10^5^ cells/well) secret IFN-γ following overnight incubation with ovarian and breast cancer cell lines (10^5^ cells/well) expressing different levels of surface αFR. Mean IFN-γ concentration ± SEM (pg/ml) from triplicate cultures is shown. **B.** Correlation between αFR expression (mean fluorescence intensity; MFI) on tumor cells and the production of IFN-γ by C4-27z CAR T cells cocultured with these tumor cells was plotted. **C.** Three primary ovarian cancer solid tumor samples (PT#1909, 1911, and 1913) and two ascites samples (PT#1801 and 1888) gated on CD45- EpCAM+ cells were stained with anti-αFR antibody (open histogram) or matched isotype controls (filled gray histogram) and analyzed by flow cytometry. A1847 and C30 cell lines served as αFR positive and negative expression controls, respectively. **D.** C4-27z CAR-modified T cells secrete IFN-γ during overnight culture with CD45-depleted αFR-expressing primary human ovarian cancer samples and αFR^pos^ control cell line A1847, but not αFR^neg^ C30 ovarian cancer cells. **E.** Antigen-specific T-cell induction of surface CD137 (4-1BB) expression was measured by FACS detection on C4-27z CAR T cells when stimulated overnight with αFR+ tumor cells (SKOV3, A1847 or T47D) but not αFR^neg^ cells. UNT T cells did not upregulate CD137 expression. CD137 expression was preferentially increased on C4 CAR+ T-cell populations but not C4 CAR-T cells, after stimulation with αFR^pos^ tumor cells. Representative dotplots from flow-cytometric analysis of CAR T cells stimulated with SKOV3 αFR^pos^ tumor cells are shown and results were similar using all αFR^pos^ tumor cells tested.

To determine whether C4 CAR-modified human T cells were able to recognize αFR^pos^ tumor cells, the C4-27z CAR-bearing T cells were cultured with tumor cells, and IFN-γ and IL-2 responses measured by ELISA. Since ovarian cancers and breast cancers frequently express αFR, a panel of established human ovarian cancer cell lines (SKOV3, A1847, OVCAR-5, OVCAR-3 and A2780) and breast cancer cell lines (SKBR3, MCF7, MDA-468 and MDA-231) that expressed surface αFR at varying levels or not at all (C30) was assembled for functional assays. As shown in Figure 2A and in Supplementary Figure 1A, C4-27z CAR T cells produced significant amounts of IFN-γ and IL-2 after coculture with all αFR^pos^ cancer cell lines, but not when cultured with αFR^neg^ cells, indicating that C4 CAR T cells functionally recognize αFR^pos^ tumor cells. The amount of IFN-γ secreted correlated with the level of surface αFR expressed by tumor cells (*R^2^* = *0.9876*, Figure 2B). In contrast, UNT T cells did not show any significant response to stimulation with αFR^pos^ tumor cells (Figure 2A).

To investigate whether C4 CAR T cells recognize primary ovarian cancer cells expressing αFR, we used cryopreserved primary ovarian cancer samples as targets. As shown in Figure 2C, CD45^neg^ EpCAM^+^ primary tumor cells in all five independent samples tested express moderate to high level αFR on cell surface. In co-culture experiments, C4-27z CAR T cells secreted substantial amounts of IFN-γ denoting T-cell activation (Figure 2D). All samples were recognized by C4-27z CAR T cells, though the amount of IFN-γ secreted did not correlate with the level of surface αFR expressed by primary tumor cells. This is likely attributed to the relatively similar levels of αFR expressed among these difference biospecimens. No IFN-γ production was detected when C4 CAR T cells were cultured with αFR^neg^ targets (C30) or from cocultures with UNT T cells, ruling out the possibility that observed reactivity was a product of allo-recognition. Moreover, CD19 CAR T cells did not produce IFN-γ when stimulated with primary αFR^pos^ cancer cells (Figure 2D), illustrating the requirement for antigen specificity for CAR-T cell activity.

We recently demonstrated that tumor-reactive human T cells from patients with cancer express the costimulatory molecule CD137 (4-1BB) in an activation-induced manner allowing for the rapid identification of recently antigen-activated T cells *ex vivo* [23]. Following incubation of C4-27z CAR T cells or UNT T cells with αFR^pos^ and αFR^neg^ tumor cells, we found robust upregulation of CD137 expression by T cells only when C4 CAR T cells were incubated with αFR^pos^ tumor cells (Figure 2E). Notably, within C4 CAR T cell/αFR^pos^ tumor cell cocultures, CD137 expression was restricted to human T cells bearing C4 CAR (Figure 2E). The CAR-negative T cell subset did not express CD137, confirming that CD137 upregulation was dependent upon specific antigen recognition by CAR T cells.

### C4 CAR T cells have anti-tumor activity *in vitro* and *in vivo*

**Figure 3:**
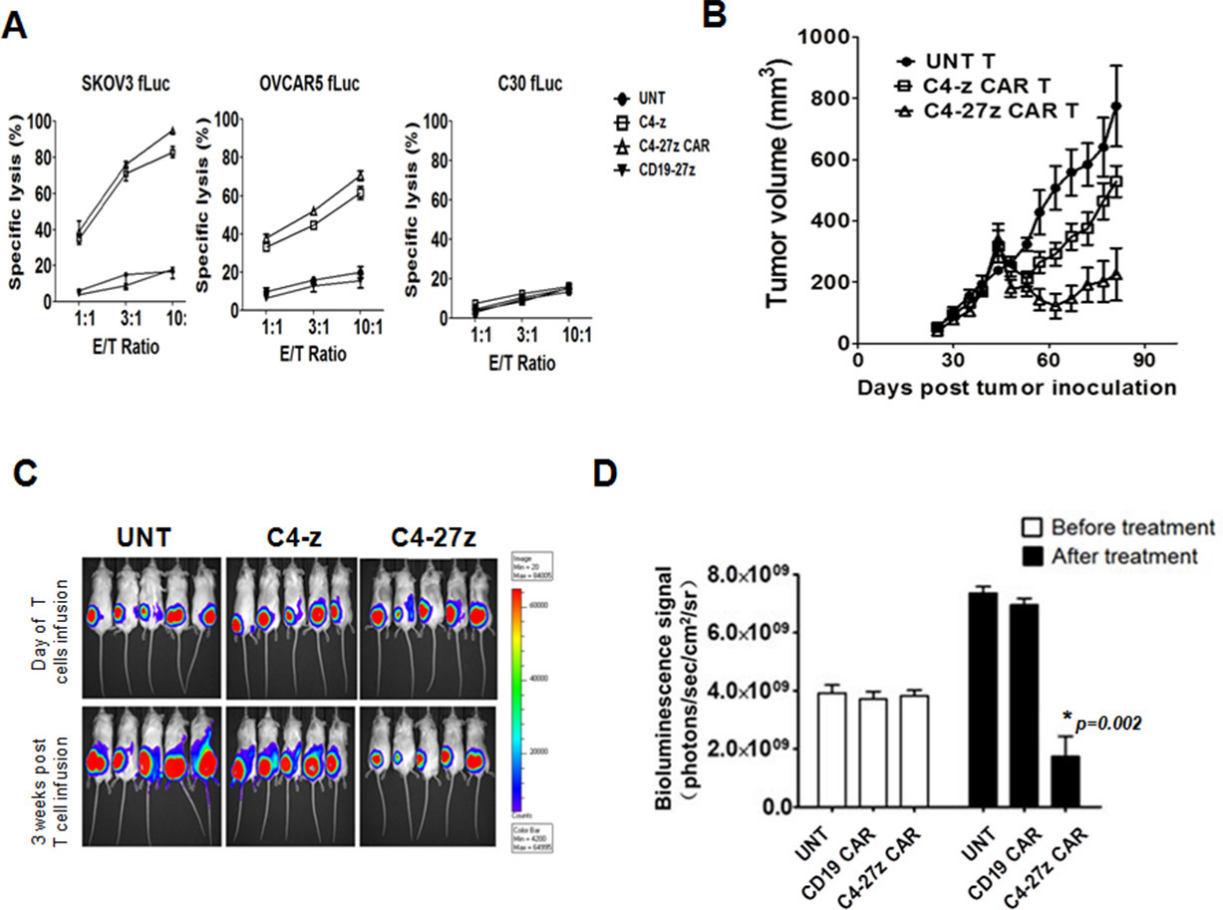
Antitumor activity of human T cells expressing C4 CAR *in vitro* and *in vivo* **A.** Cytotoxicity of αFR-expressing tumor cells SKOV3 (higher expression) and OVCAR5 (lower expression) by CAR T cells in 18-hour bioluminescence assay at the indicated E/T ratio. UNT T cells and CD19-27z CAR T cells served as negative effector controls. C30 cells served as negative target cell control. Percent tumor cell viability was calculated as the mean luminescence of the experimental sample minus background divided by the mean luminescence of the input number of target cells used in the assay minus background times 100. All data are represented as a mean of triplicate wells. **B.** NSG mice bearing established s.c. tumor were treated with intravenous (i.v.) injections of 1 × 10^7^ CAR+ T cells on day 40 post tumor inoculation. Tumor growth was assessed by caliper measurement [*V* = 1/2(length × width^2^)]. **C.** Tumor progression was also followed by *in vivo* bioluminescence imaging. **D.** NSG mice received i.p. injection of 3 × 10^6^ SKOV3 fLuc tumor cells and were randomized into 3 groups of 5 mice each before beginning therapy with UNT T cells or T cells expressing C4-27z or CD19-27z CAR via i.v. infusion on day 21 and 25 after tumor inoculation. Photon emission from fLuc tumor cells was quantified and the mean ± SD bioluminescence signal determined prior to and two weeks after second i.v. injection of 1 × 10^7^ CAR-T cells on days 21 and 25 after tumor inoculation.

We next evaluated the cytolytic potential of αFR-specific C4 CAR-T cells *in vitro* in an overnight luminescence assay using firefly luciferase (fLuc+)-expressing cancer cells as targets. C4-z and C4-27z CAR T cells specifically and efficiently lysed the αFR^pos^ human ovarian cancer cell lines SKOV3 (higher expression) and OVCAR5 (lower expression), but not αFR^neg^ C30 cells (Figure 3A) at effector to target ratios of 10:1 to 1:1. Control CD19 CAR T cells did not lyse ovarian cancer cells, which lack CD19.

To assess the antitumor activity of C4 CAR T cells against αFR^pos^ human tumor *in vivo*, we first evaluated their potency in a xenograft mouse model of subcutaneous ovarian cancer. Immunodeficient nonobese diabetic/severe combined immunodeficient/IL-2γc^null^ (NSG) mice were inoculated subcutaneously with fLuc+ αFR^pos^ human ovarian cancer SKOV3 cells on the hind flank and received intravenous injection of 10^7^ CAR+ T cells on day 40 after tumor inoculation when tumors were ∼250 mm^3^ in size and evident by bioluminescence imaging. Intravenous administration of C4-27z CAR-T cells significantly delayed tumor progression compared to tumor growth observed in control mice treated with UNT T cells (*p* = 0.005). Furthermore, incorporation of CD27 signals enhances antitumor activity *in vivo*, because C4-27z T cells therapy was superior to therapy with the first-generation C4-z CAR T cell (*p* = 0.015), which lacks a CD27 costimulation domain (Figure 3B, 3C).

Advanced ovarian cancer usually presents with widespread intra-abdominal metastasis, resulting in malignant ascites. A xenogeneic mouse model of advanced intraperitoneal (IP) metastatic cancer was established to evaluate the functional activity of C4-27z CAR T cells against tumor localized to a more physiologically relevant compartment. In this model, untreated mice develop multiple nodular peritoneal tumors and ascites, with tumors also found on the surfaces of the diaphragm, intestines uterus and associated fat, and stomach, requiring euthanasia approximately 40 days post-inoculation. We injected 3 × 10^6^ fLuc+ SKOV3 cells IP into NSG mice and followed tumor growth by bioluminescence imaging (BLI). After 3 weeks, mice received intravenous injections of 10^7^ C4-27z CAR+ T cells given on day 21 and day 25 post tumor inoculation. Control groups of tumor-bearing mice were injected with untransduced or CD19-27z CAR T cells, and in these mice, the tumors grew rapidly, as measured by BLI (Supplementary Figure 1B). In contrast, there was a significant difference in tumor burden between C4-27z CAR T cells and control T-cell groups 14 days after second T-cell dose injection (*p* = 0.002; Figure 3D). CD27 costimulated CD19 CAR T cell infusion elicited no antitumor activity, thus we conclude that antitumor activity mediated by C4-27z CAR T cells is also antigen-specific *in vivo*.

### Enhanced function of the human C4 CAR compared to murine MOv19 CAR *in vitro*

**Figure 4:**
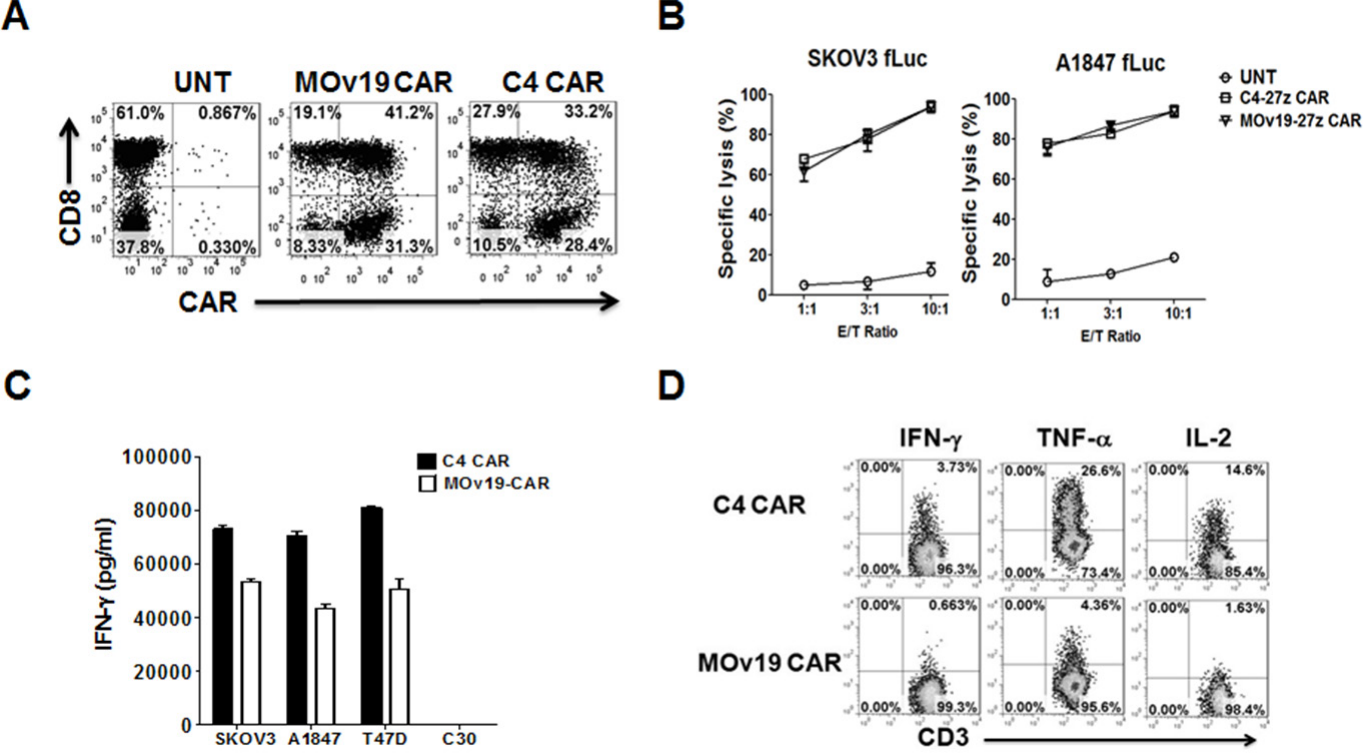
Comparison of anti-tumor activity of αFR-specific C4 and MOv19 CARs with CD27 costimulatory endodomain *in vitro* **A.** C4 and MOv19 CARs expression on primary human T cells can be detected via biotin-labeled recombinant αFR protein followed by SA-PE. As shown, both CD8+ T and CD8- (CD4+) cells can efficiently express CARs as measured by flow cytometry. **B.** C4 and MOv19 CARs-transduced T cells showed lytic function in a bioluminescent killing assay. CAR-T cells killed αFR+ SKOV3 and A1847 at the indicated E/T ratio after more than 20 hours. UNT T cells served as negative controls. Mean and SD of triplicate wells from 1 of at least 3 independent experiments is shown. **C.** C4 or MOv19 CAR T cells were co-cultured with αFR+ target cells (SKOV3, A1847 and T47D) and αFR- (C30) at a 1:1 E:T ratio. **D.** C4 or MOv19 CAR T cells were stimulated with SKOV3 cells for 5-hour in the presence of Golgi inhibitor and analyzed by flow cytometry for intracellular IFN-γ, TNF-α and IL-2. Results are shown for representative donor T cells and are reproducible in multiple donors (*n* = 3).

Using the same production and concentration protocols in parallel, we found that the C4 CAR-encoding lentivirus has a higher effective titer than the murine MOv19 CAR, possibly the result of more efficient expression of the human scFv on human T cells (Supplementary Figure 2A). Indeed, we observed a multiplicity of infection (MOI) of C4 CAR lentivirus as low as 1 is sufficient to infect > 20% human T cells, while the MOv19 CAR lentivirus required a MOI of 5 (Supplementary Figure 2B). Thus, for the following experiments, T cells were infected with a MOI of 2 and 5 of concentrated C4-27z and MOv19-27z vector, respectively, and both C4 and MOv19 CAR surface expression on T cells were detected via recombinant αFR protein staining (Figure 4A).

ScFvs used for CAR construction require a minimal antigen affinity to achieve activation threshold for the engineered T cell, however, higher affinity scFvs do not necessarily induce a more potent activation of CAR T cells than low affinity scFvs [24]. Since the binding affinity of the C4 Fab fragment (2 × 10^7^ M^−1^) is approximately five-fold weaker than that of the MOv19 antibody [21], we examined whether the potentially lower affinity of the C4 scFv used to construct the fully human C4 CAR might influence redirected T-cell function via comparison to the MOv19 CAR containing a potentially higher affinity anti-αFR scFv. T cells modified to express either the C4-27z or MOv19-27z CAR specifically lysed αFR^pos^ SKOV3 and A1847 tumor cells with approximately equivalent efficiency in overnight co-cultures (Figure 4B). However, *in vitro* cytokine production analysis showed that MOv19-27z CAR T cells secreted significantly less IFN-γ than C4 CAR T cells at an equivalent 1:1 E:T ratio after overnight co-culture (Figure 4C). This result was validated by 5-hour intracellular cytokine production assays. Representative fluorescence activated cell sorter (FACS) plots of 5-hour intracellular cytokine expression by tumor-activated CAR T cells show that both C4 and MOv19 CAR T cells produce IFN-γ, TNF-α and IL-2 cytokines when incubated overnight with αFR^pos^ SKOV3 ovarian cancer cells, but MOv19 CAR T cells produced less of these cytokines than C4 CAR T cells (Figure 4D). The frequency of C4 CAR T cells expressing cytokine was 5.6-fold higher for IFN-γ, 6.1-fold for higher TNF-α and 9-fold higher for IL-2, than that observed in MOv19 CAR T cells *in vitro*. UNT T cells cocultured with αFR^pos^ or αFR^neg^ cancer cells (not shown), or CAR T cells cocultured with αFR^neg^ cancer cells, did not produce proinflammatory cytokines (Supplementary Figure 3A and 3B).

**Figure 5:**
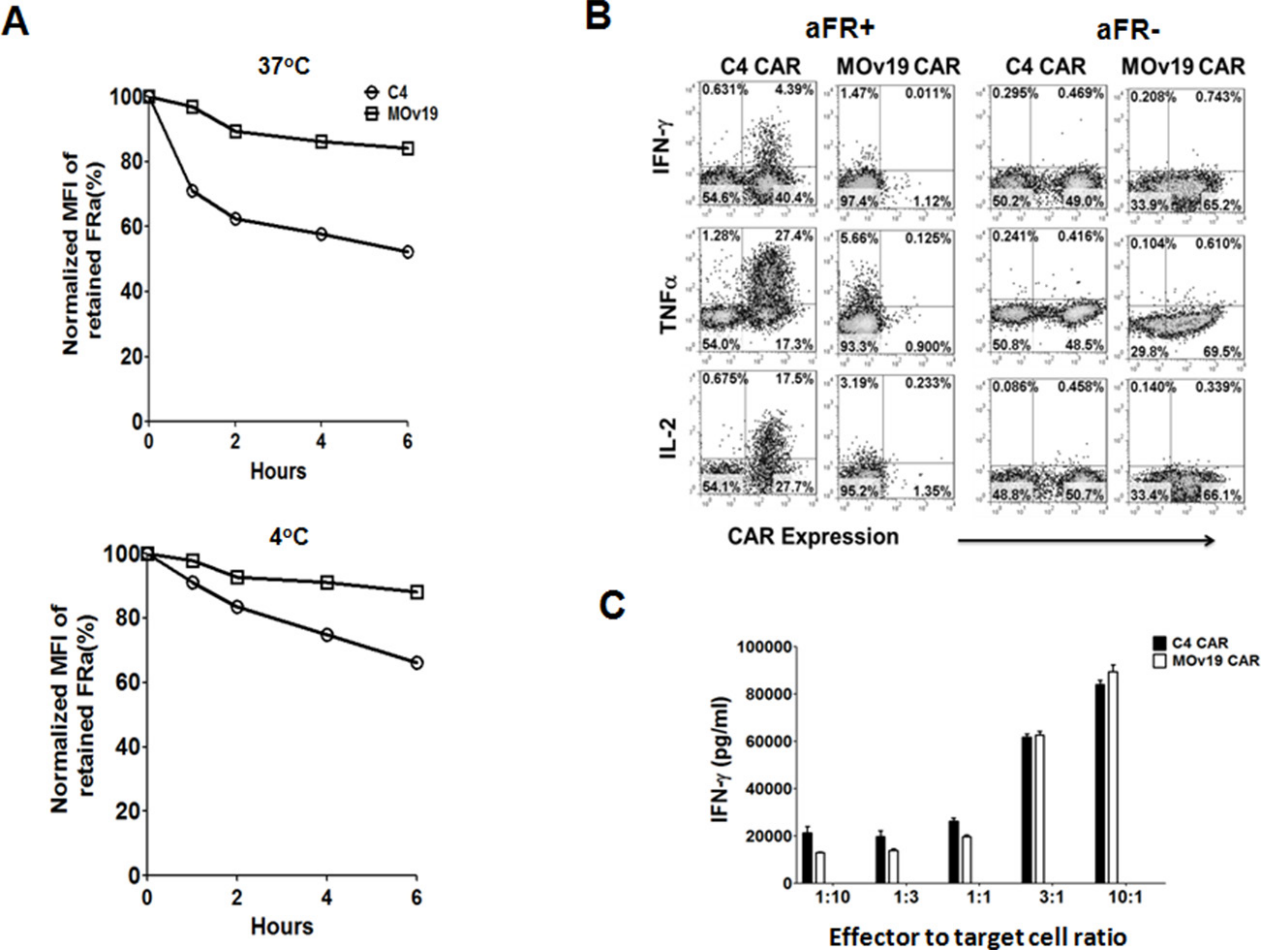
CAR down-modulation may impair the antitumor activity of MOv19 CAR but not C4 CAR **A.** αFR dissociation assay. C4-27z or MOv19-27z CAR T cells were labeled with recombinant biotinylated αFR protein and then incubated at 37°C (Upper) or 4°C (Lower) in a time course assay in the presence of a ten-fold excess of nonbiotinylated αFR. Antigen retention on the cell surface was assessed by flow cytometry by adding SA-PE after the end of each culture period. Percent retained αFR (y-axis) was normalized and scored as mean fluorescence intensity (MFI) postincubation ÷ preincubation MFI × 100. **B.** C4 and MOv19 CAR T cells were stimulated with SKOV3 or C30 cells for 5-hour in the presence of Golgi inhibitor and analyzed by flow cytometry for T cell surface of CAR expression and intracellular IFN-γ, TNF-α and IL-2. **C.** IFN-γ release assay of C4 and MOv19 CAR T cells after overnight co-culture with αFR+ tumor cells at 1:10, 1:3, 1:1, 3:1 and 10:1 E:T ratios. Results are shown for representative donor T cells and are reproducible across multiple donors (*n* = 3).

Our results *in vitro* suggested that the fully human C4 CAR T cells may be functionally superior to MOv19 CAR T cells. To understand the mechanisms accounting for reduced function by MOv19 CAR T cells, we carefully measured for relative binding to recombinant αFR protein, antigen-induced cell death (AICD) and CAR expression on T cells after co-incubation with antigen-expressing tumor cells. First, C4 and MOv19 CAR T cells were pre-loaded with biotin labeled recombinant αFR protein, and measured for surface protein dissociation over time at either 4 or 37°C in the presence of excess non-biotinylated αFR competitor. Within one hour, less αFR protein was detectable on the surface of C4 CAR T cells, in comparison to MOv19 CAR, at either temperature and the level of dissociation was dependent on both time and temperature, and was higher in C4 CAR T cells under all conditions (Figure 5A). Similar results were obtained in titration analysis on the binding of biotinylated αFR protein to MOv19 and C4 CAR T cells (Supplementary Figure 3C), suggesting that C4 in the CAR construct had a lower affinity for soluble αFR antigen than MOv19 CAR. We have shown previously that supraphysiological expression of antigen on the target cell surface can induce antigen induced cell death of CAR T cells [25], however, the frequency of apoptotic cells in C4 CAR or MOv19-CAR T cells after stimulation with SKOV3 cells was similar, as measured by Annexin V+/7-AAD+ staining (Supplementary Figure 4A and 4B), suggesting that AICD did not play a major role. Lastly, stimulation with SKOV3 cancer cells, which express a high level of αFR, induced a rapid and marked down-modulation of surface MOv19 CAR expression following antigen engagement (Figure 5B). Five hours after exposure to tumor cells, MOv19 CAR frequency was rapidly decreased from about 65% of T cells to ∼1%. This finding was additionally confirmed by using αFR^pos^ A1847 cells and the breast cancer cell line T47D, which also express high levels of αFR (Supplementary Figure 4C). By comparison, the C4 CAR was not markedly down-modulated (Figure 5B) and intracellular cytokine expression analysis showed that T cells with maintained C4 CAR surface expression produced IFN-γ, TNF-α and IL-2, while cytokine production was exclusively detected in the CAR-negative fraction of the MOv19 group, indicating that CAR down-modulation and cytokine production had occurred following antigen encounter.

Given differences in relative affinity, we hypothesized that overall avidity between CAR and target molecule might account for the observed difference in CAR expression. We therefore evaluated the impact of T cell to target cell ratio on relative CAR expression by C4 or MOv19 CAR T cells following co-culture with SKOV3 cells. At lower E:T ratios of 1:10, 1:3 and 1:1, MOv19 CAR T cells showed a marked, dose-dependent down-modulation in CAR expression compared with C4 CAR, which maintained ∼50% of initial CAR expression at the lowest E:T ratio tested (Supplementary Figure 5). However, at high E:T ratios of 3:1 and 10:1 where tumor antigen is more limiting, T cells bearing either C4 or MOv19 CAR maintained high CAR expression. Consistent with changes in CAR expression after antigen stimulation, C4 CAR T cells released more IFN-γ than MOv19 CAR T cells at E:T ratios of 1:10, 1:3 and 1:1, but similar amounts at E:T ratios of 3:1 and 10:1 (Figure 5C). Thus, CAR down-modulation occurs in an antigen dose-dependent fashion with anti-αFR CAR T cells bearing the higher affinity MOv19 scFv being more sensitive to low antigen level.

### Comparable antitumor activity of C4 and MOv19 CAR T cells *in vivo*

**Figure 6:**
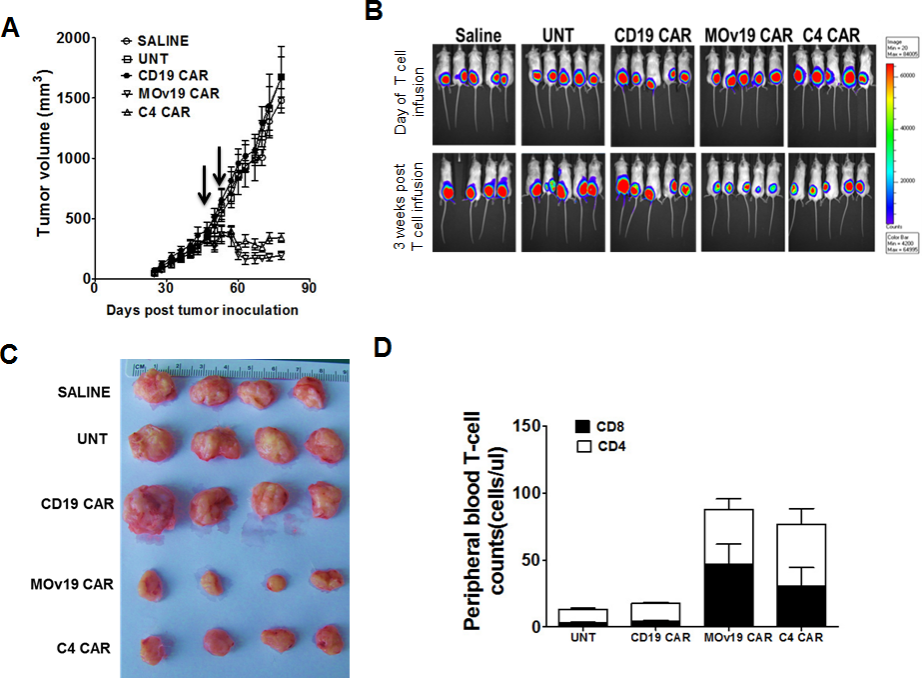
Antitumor activity of C4-CAR T cells is comparable to MOv19 CAR T cells **A.** Tumor regression mediated by C4-27z and MOv19-27z CAR T cells. NSG mice bearing established subcutaneous tumor were treated with i.v. injections of 1 × 10^7^ C4-27z and MOv19-27z CAR+ T cells or control CD19-27z and UNT T cells or saline on day 40 and 45. Tumor growth was assessed by caliper measurement. Tumors treated with C4-27z CAR or MOv19 CAR T cells (∼60% CAR expression) regressed (arrows indicate days of T cell infusion); tumors treated with saline, UNT or CD19-27z CAR T cells did not regress 3 weeks post-first T cell dose. **B.** SKOV3 fLuc+ bioluminescence signal was decreased in C4-27z and MOv19-27z CAR T cells treated mice compared with the CD19-27z and the control treatment groups 3 weeks after the first T cell dose. **C.** Macroscopic evaluation of resected tumor specimens following T cell therapy. Tumors were harvested from mice at the time of euthanasia, nearly 45 days after first T cell injection. **D.** Stable persistence of C4 CAR and MOv19 CAR T cells *in vivo.* Peripheral blood was collected 3 weeks after the first T cell infusion and quantified for the absolute number of human CD4+ and CD8+ T cells/μl of blood. Mean cell count ± SEM is shown with *n* = 5 for all groups.

To compare the antitumor capacity of C4 CAR T cells with MOv19 CAR T cells *in vivo*, NSG mice with large, established subcutaneous SKOV3 tumors (∼300 mm^3^) received intravenous injections of 10^7^ CAR+ T cells on days 40 and 47 post-tumor inoculation. Tumors in animals treated with saline, UNT or CD19-27z CAR T cells continued to grow rapidly. In contrast, mice receiving C4-27z or MOv19-27z CAR T cells experienced tumor regression (*p* < 0.0001), compared with all 3 control groups at the latest evaluated time point. The antitumor activity of MOv19-27z CAR T cells appeared slightly better than that of C4-27z CAR T cells, but were statistically similar even at the final time point (*p* = 0.058; Figure 6A). BLI of tumor xenografts before and 3 weeks after T cells injection showed progressive growth of tumors in all animals receiving control T cells but not in CAR T cells groups (Figure 6B). Tumor BLI results were consistent with the size of resected residual tumors (Figure 6C). Next, we analyzed the persistence of transferred T-cells in the peripheral blood 3 weeks following adoptive transfer and detected higher numbers of CD4+ and CD8+ T cells in mice treated with both the C4 and MOv19 CAR T cells groups compared with the UNT and CD19-27z CAR T cells treatment group (Figure 6D), suggesting that tumor antigen recognition drives the survival of the adoptively transferred T cells *in vivo*. These results demonstrated that the anti-tumor activity of the C4 CAR is comparable to the MOv19 CAR, which was described previously [19, 20], and confirm that the C4 CAR, despite its decreased affinity, is suitable for *in vivo* application.

### Anti-αFR CAR with lower affinity may decrease the risk of “on-target” toxicity

**Figure 7:**
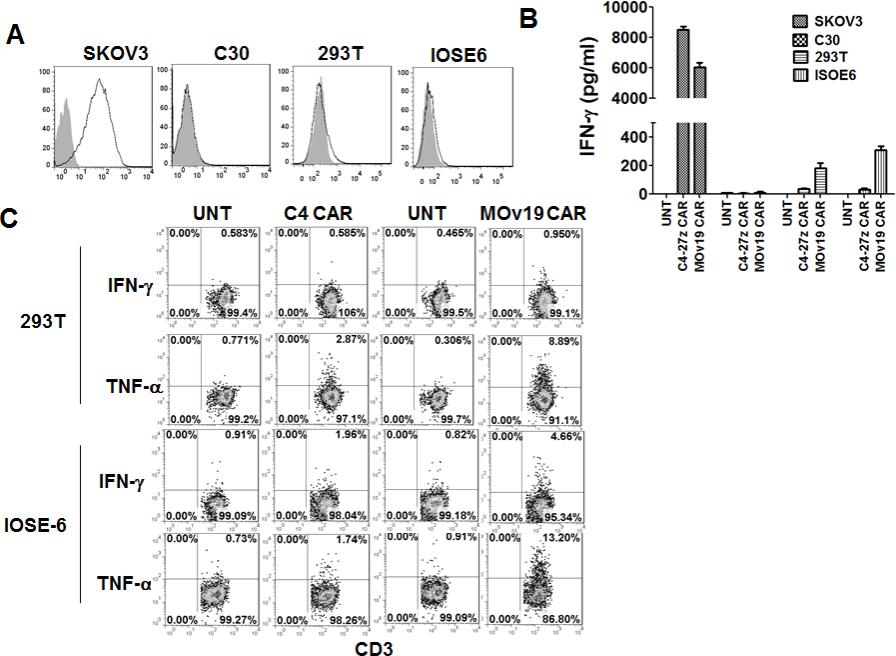
C4 CAR T cells showed minimal cytokine release *in vitro* **A.** Human embryonic kidney 293T cells and normal epithelial ovarian cell line IOSE6 express very low level of αFR. SKOV3 and C30 served as positive and negative controls, respectively. **B.** C4-27z CAR T cells secret minimal amount of IFN-γ following overnight incubation with normal 293T cells and IOSE 6 cell lines expressing low levels of surface αFR compared to MOv19-27z CAR T cells. **C.** C4 and MOv19 CAR T cells were stimulated with 293T or IOSE6 cells for 5-hour in the presence of Golgi inhibitor and analyzed by flow cytometry for intracellular IFN-γ and TNF-α.

On-target toxicities have been observed in clinical trials [26–28] with CAR T-cells specific for tumor associated antigens that are expressed at low levels on normal cells, and a critical issue to be addressed is whether CARs with higher affinity may increase the risk of toxicity. In a report by Chmielewski *et al*., higher affinity CARs specific for HER-2 antigen exhibited less discrimination between target cells with high or low Ag expression levels [24]. To investigate the functional effect of exposure of primary human T cells modified with C4 CAR or MOv19 CAR to normal cells expressing low levels of αFR, we analyzed cytokine production of C4 CAR and MOv19 CAR T-cells after co-culture with human embryonic kidney 293T cells or normal epithelial ovarian cell line IOSE 6, which express low but detectable levels of αFR, and αFR^pos^ SKOV3 cells (Figure 7A). C4 and MOv19 CAR T cells responded against SKOV3 with greater activity observed again from C4 CAR T cells. However, greater IFN-γ cytokine production was observed from the MOv19 CAR T cells in response to low antigen expressing cells, suggesting that MOv19 CAR T cells are more functionally avid and sensitive to low antigen (Figure 7B). Similar to what we observed in overnight IFN-γ release assays, 5-hour intracellular cytokine secretion assays showed that more MOv19 CAR T cells produced IFN-γ and TNF-α in response to low antigen on normal cells (Figure 7C), which is one primary proposed contributor to the “on-target” cytokine storm [26], as compared with C4 CAR T cells. These data suggest that the newly described C4 CAR may have a more appropriate affinity for the delivery of safe and effective engineered T cell therapy of cancer.

## DISCUSSION

αFR is an attractive target for cancer immunotherapy due to its overexpression on the surface of epithelial ovarian cancer and many other epithelial cancers. αFR is expressed at low levels in some normal tissues, however its expression is largely restricted to the apical surface of some polarized epithelial cells of normal tissues [16], where it is inaccessible to circulating drugs. This suggests that a “druggable window” may exist for the creation of safe, effective therapy. We have recently constructed a murine MOv19 scFv-based αFR-specific CAR that overcomes the limitations of past CAR approaches by improving the persistence and anti-tumor activity of transferred CAR T cells *in vivo* [19, 20]. Here, we constructed a novel fully human anti-FR C4 CAR and addressed the potential issue of CAR transgene immunogenicity and risk for toxicity. We observed that C4 CAR-modified T cells have *in vitro* activity against ovarian cancer and breast cancer cells and *in vivo* activity against αFR expressing ovarian cancer.

T-cell based targeting of αFR has been tested in patients with advanced ovarian cancer with encouraging results. Kershaw and colleagues [17] transferred T cells that were retargeted to αFR by a first-generation MOv18 scFv-based CAR to immunocompetent patients with advanced ovarian cancer. Therapy using MOv18-z CAR was safe and feasible; however, no patient experienced a tumor response which was attributed to a lack of transferred T-cells persistence after infusion, poor tumor localization, and the development of a human anti-mouse antibody (HAMA) that reduced CAR T-cell activity in *in vivo* study. Unlike previous MOv18 and MOv19 CARs that utilize scFvs derived from mouse monoclonal antibodies, the fully human anti-αFR C4 CAR is predicted to be less likely to induce xenogeneic HAMA or T cell responses in humans, which have limited their impact *in vivo*.

The therapeutic success of adoptive therapy with CAR T cells depends on the appropriate costimulation of CD3z to induce full T-cell activation. These costimulatory cytoplasmic signaling domains derived from the T-cell costimulatory molecules CD28 [29–31], 4-1BB (CD137) [20, 30], OX40 (CD134) [30], ICOS [32] or CD27 [19]. CD27 is a member of the TNF receptor family, which is also known as a T cell costimulatory molecule. This work builds upon our previous study which showed CD27 costimulation promotes CAR-T cell survival and antitumor function *in vivo* [19]. More recently, Duong et al. [33] demonstrated that HER2 specific CAR T cells bearing the DAP10, CD27 and CD3z inhibited tumor growth to a greater degree than T cells expressing the CD28z CAR, suggesting an important role of CD27 costimulation. Consistent with previous study [19], CD27-bearing C4 CAR-T cells facilitated superior regression of established tumors in a xenograft model of subcutaneous human ovarian compared with the marginal inhibition achieved with conventional, non-costimulated CAR-T cells. In addition, CD27-bearing C4 CAR-T cells significantly controlled the tumor growth in metastatic intraperitoneal human ovarian cancer model. Notably, T cells modified to express an irrelevant anti-CD19 CAR were unable to alter tumor growth demonstrating the high specificity of the CAR system and ruling out the possibility of xenogeneity as the source of the tumor response.

The C4 CAR expressed on the T cell surface exhibited reduced association and increased dissociation of αFR protein, relative to MOv19 CAR T cells. The decreased affinity of the fully human C4 CAR could affect T-cell recognition. However, a direct comparison of cytokine production after tumor engagement by T cells modified with the C4 and MOv19 CARs showed that the C4 CAR with lower affinity was superior in conditions of high antigen exposure. This may be due to the rapid internalization of the higher affinity MOv19 CAR upon encountering high levels of antigen. With lower antigen availability, the anti-tumor activity of C4 CAR T cells is more comparable to MOv19 CAR T cells. It would be interesting to determine the optimal ratio of CAR T cells and tumor cells necessary to enhance CAR T cell function *in vitro* and *in vivo*; this would likely require quantitative analysis using mathematical model described previously [34]. In comparing relative antitumor activity *in vivo*, we found that T cells expressing the high-affinity MOv19 CAR mediated slightly superior activity *in vivo* compared with the C4 CAR. However, this difference is not statistically significant, suggesting that the affinity of C4 CAR is adequate for *in vivo* application.

Possible on-target, off-tumor toxicities resulting from the expression of TAAs on normal tissues need to be considered in the application of CAR approach. The development of high affinity CAR or TCR with great anti-tumor activity can lead to severe toxicity [26], [35]. C4 CAR T cells release less cytokine compared with MOv19 CAR T cells when encountering normal cells expressing low levels of αFR, yet retained enhanced reactivity against cancer cells. Thus, the relative lower affinity of C4 CAR could theoretically decrease the risk of on-target toxicity, while the higher affinity MOv19 CAR may increase this risk *in vivo*. However, the potential for off-target toxicity in the clinical setting is not precluded by these studies. To this end, one may utilize an RNA electroporation approach that results in transient CAR expression on electroporated T cells to provide a safer platform in the clinical setting. Moreover, it should be possible to tune scFv affinity by mutagenesis *in vitro* if it was safe in early clinical trials.

In consideration of clinical application, there are several advantages to the use of fully human C4 CAR T cells over murine MOv18 or MOv19 CAR T cells as a therapy for αFR expressing cancer. The first advantage is that it is likely to be less immunogenic than murine scFv format upon application for adoptive T cell immunotherapy in patients. The immunogenicity of mouse transgenes is noted in numerous trials of adoptive immunotherapy using autologous T cells modified to express mouse-derived scFvs or tumor antigen-specific TCRs [17, 36, 37], which limited the persistence and function of the transferred gene-modified cells. The second advantage is that C4 CAR can be more easily expressed on human T cell surface compared with MOv19 CAR when using the same MOI for each vector. These results suggest a possible significant cost savings for clinical grade lentivirus production. The third advantage is that the human C4 CAR with decreased affinity may release lower levels of proinflammatory cytokines upon encounter with healthy cells that express low levels of αFR while still retaining comparable antitumor activity to MOv19 CAR T cells *in vivo*. In conclusion, our study shows that fully human C4-27z CAR T cells destroy αFR-positive ovarian cancer and breast cancer cells *in vitro* and mediate ovarian cancer regression *in vivo*. These data provide the rationale for the clinical investigation of C4 CAR T-cell therapy of a wide spectrum of αFR expressing epithelial malignancies.

## MATERIALS AND METHODS

### Cell lines

Lentivirus packaging was performed in the immortalized normal fetal renal 293T cell line purchased from ATCC. Human cell lines used in immune based assays include the established human ovarian cancer cell lines SKOV3, A1847, T47D and C30. The human lymphoid cell lines SUP-T1 was used for lentivirus titer analysis. For bioluminescence assays, target cancer cell lines were transfected to express firefly luciferase (fLuc). The mouse malignant mesothelioma cell line, AE17 (kindly provided by Steven Albelda, University of Pennsylvania) was used as negative control. All cell lines were maintained in R10 medium: RPMI-1640 supplemented with 10% heat inactivated FBS, 100U/mL penicillin, 100mg/mL streptomycin sulfate, 10mmol/L HEPES).

### CAR construction and lentivirus production

The pHEN2 plasmid containing the anti-αFR C4 scFv kindly provided by Dr. Silvana Canevari was used as a template for PCR amplification of a 729-bp C4 fragment using the following primers: 5′-ataggatcccagctggtggagtctgggggaggc-3′ (BamHI is underlined) and 5′-atagctagcacctaggacggtcagcttggtccc-3′ (NheI is underlined). Third generation self-inactivating lentiviral expression vectors pELNS previously described were digested with BamHI and NheI and gel purified. The digested PCR products were then inserted into the pELNS vector containing CD3z or CD27-CD3z T cell signaling domains in which transgene expression is driven by the elongation factor-1α (EF-1α) promoter. The resulting construct was designated pELNS-C4-z or C4-27z. High-titer replication-defective lentiviral vectors were produced and concentrated as previously described [31]. Briefly, 293T cells were seeded in 150 cm^2^ flask and transfected using Express In (Open Biosystems) according to manufacturer's instructions. Fifteen micrograms of αFR-specific CAR transgene plasmid were cotransfected with 7 ug pVSV-G (VSV glycoprotein expression plasmid), 18 ug pRSV.REV (Rev expression plasmid) and 18 ug pMDLg/p.RRE (Gag/Pol expression plasmid) with 174 ul Express In (1 ug/ul) per flask. Supernatants were collected at 24 h and 48 h after transfection, concentrated 10-fold by ultracentrifugation for 2 hours at 28, 000 rpm with a Beckman SW32Ti rotor (Beckman Coulter). The viruses were aliquoted into tubes and stored at −80°C until ready to use for titering or experiments. All lentiviruses used in the experiments were from concentrated stocks.

### Determination of lentiviral titer

Titers of concentrated lentiviral vectors encoding αFR CAR were determined by serially (3-fold) diluting vector preparations in R10 medium and transduce SUP-T1 cells. Briefly, SUP-T1 cells (20,000 cells/100 ul/well) were seeded in a single well of a 96-well plate and 50 ul 3-fold diluted vector supernatant was transferred and incubated overnight. The next day, feed the cells with 100 ul pre-warmed R10 medium. Two days post transduction, vector titers were determined by flow cytometry applying standard flow cytometric methods for analysis of CAR expression. The titers (transducing units [TU] (% positive/100) × 2E4 × 20 × dilution. All the experiments were repeated at least three times and average titers obtained from the experiments were used for data analysis.

### Human T cells and transfection

Primary human CD4+ and CD8+ T cells, purchased from the Human Immunology Core at University of Pennsylvania, were isolated from healthy volunteer donors following leukapheresis by negative selection. All specimens were collected under a protocol approved by a University Institutional Review Board, and written informed consent was obtained from each donor. T cells were cultured in R10 medium and stimulated with anti-CD3 and anti-CD28 monoclonal antibodies (mAb)-coated beads (Invitrogen). Eighteen to 24 hours after activation, human T cells were transduced using a spinoculation procedure. Briefly, 0.5 × 10^6^ T cells were infected with a multiplicity of infection (MOI) of 2 and 5 of concentrated C4-27z and MOv19-27z vector, respectively. Mixtures of cells and vectors were centrifuged at room temperature for 90min (2,500 rpm) in a table-top centrifuge (Sorvall ST 40). Human recombinant interleukin-2 (IL-2; Novartis) was added every 2–3 days to a 100 IU/mL final concentration and a cell density of 0.5 × 10^6^ to 1 × 10^6^ cells/mL was maintained. Once engineered T-cell cultures appeared to rest down, as determined by both decreased growth kinetics and cell-sizing determined using the Multisizer 3 Coulter Counter (Beckman Coulter), the T cells were used for functional analysis.

### Flow cytometric analysis

The following monoclonal antibodies were used for phenotypic analysis: APC-Cy7 anti-human CD3; FITC antihuman CD4; APC anti-human CD8; PE-anti-human CD45; PE anti-human CD137. 7-Aminoactinomycin D (7-AAD) was used for viability staining. All monoclonal antibodies were purchased from BD Biosciences. In T cell transfer experiments, peripheral blood was obtained via retro-orbital bleeding and stained for the presence of human CD45, CD4, and CD8 T cells. After gating on the human CD45+ population, the CD4+ and CD8+ subsets were quantified using TruCount tubes (BD Biosciences) with known numbers of fluorescent beads as described in the manufacturer's instructions. Tumor cell surface expression of αFR was performed using MOv18 mAb followed by APC-labeled goat anti mouse Ab. T cell surface expression of the both C4 and MOv19 CAR was evaluated using biotin-labeled recombinant αFR protein (R&D Systems, Inc) followed by Streptavidin-APC (eBioscience, Inc.) or biotin-labeled rabbit anti-human IgG and goat anti-Mouse IgG F(ab’)_2_ fragment followed by Streptavidin-APC, respectively. To determine cytokine production in CAR T cells, cells were cocultured with FR^pos^ ovarian cancer cells for 5 h. After surface markers were stained, cells were fixed and permeabilized using Cytofix/Cytoperm and Perm/Wash buffer (BD Biosciences) according to the manufacturer's instructions. Then cells were stained with fluorescence-conjugated cytokine antibodies including PE anti-human IFN-γ, Pacific blue anti-human TNF-α or FITC anti-human IL-2 before analysis. Flow cytometry was performed with a BD FACS Canto II flow cytometer (BD Biosciences) and flow cytometric data were analyzed with FlowJo version 7.2.5 software (Tree Star, Ashland, OR).

### Cytokine release assays

Cytokine release assays were performed by coculture of 1 × 10^5^ T cells with 1 × 10^5^ target cells per well in triplicate in 96-well flat bottom plates in a 200 ul volume of R10 medium. After 20–24 hours, coculture supernatants were assayed for presence of IFN-γ and IL-2 using an ELISA Kit, according to manufacturer's instructions (Biolegend, San diego, CA). Values represent the mean of triplicate wells.

### Cytotoxicity assays

For the cell-based bioluminescence assays, 5 × 10^4^ firefly Luciferase (fLuc)-expressing tumor cells were cultured with R10 media in the presence of different ratios of transduced T cells with the use of a 96-well Microplate (BD Biosciences). After incubation for ∼20 hours at 37°C, each well was filled with 50 uL of DPBS resuspended with 1 ul of D-luciferin (0.015 g/mL) and imaged with the Xenogen IVIS Spectrum. Percent tumor cell viability was calculated as the mean luminescence of the experimental sample minus background divided by the mean luminescence of the input number of target cells used in the assay minus background times 100. All data are represented as a mean of triplicate wells.

### Antigen dissociation assay

C4-27z or MOv19-27z CAR (both 90% CAR expression) T cell were harvested, washed once with fluorescent-activated cell sorting buffer and stained with 0.5 μg/ml biotinylated-αFR protein for 30 minutes at 4°C. Then the cells were washed two times before the addition of 5 μg/ml of nonbiotinylated αFR competitor and incubation at a 4°C or 37°C for different time points (0 < *t* < 6 hours). At indicated time points cells were removed from 4°C or 37°C, washed again, labeled with biotinylated streptavidin, washed and analyzed for percent αFR-positive and mean fluorescence intensity by flow cytometry.

### Titration analysis on the binding of αFR protein to αFR CAR T cells

Activated T cells were transduced with lentiviral vector expressing MOv19-27z or C4-27z-CAR and analyzed for CAR expression on day 14. One hundred thousand UNT or CAR T cells were stained with 0.2, 0.5, 1, 2, 5, 10, 20, 50 or 120 nM/sample of biotinylated αFR. T cells were then washed and stained with phycoerythrin (PE)-conjugated streptavidin (SA). No αFR protein T cells sample was stained with SA-PE alone.

### Xenograft model of ovarian cancer

All animals were obtained from the Stem Cell and Xenograft Core of the Abramson Cancer Center, University of Pennsylvania. Six to 12-week-old NOD/SCID/γ-chain−/− (NSG) mice were bred, treated and maintained under pathogen-free conditions in-house under University of Pennsylvania IACUC approved protocols. For an established ovarian cancer model, 6 to 12-week-old female NSG mice were inoculated s.c. with 3 × 10^6^ SKOV3 fLuc+ cells on the flank on day 0. After tumors become palpable at about 1 month, human primary T cell (CD4+ and CD8+T cells used were mixed at 1:1 ratio) were activated, and transduced as described above. After 2 weeks of T cell expansion, when the tumor burden was ∼200–300 mm^3^, mice were treated with T cells. The route, dose, and timing of T-cell injections is indicated in the individual figure legends. Tumor dimensions were measured with calipers, and tumor volumes calculated using the formula *V* = 1/2(length × width^2^), where length is greatest longitudinal diameter and width is greatest transverse diameter. Animals were imaged prior to T cell transfer and about every week thereafter to evaluate tumor growth. Photon emission from fLuc+ cells was quantified using the “Living Image” software (Xenogen) for all *in vivo* experiments. Tumors were resected immediately after euthanasia approximately 40 days after first T cell dose for size measurement and immunohistochemistry.

For the intraperitoneal model of ovarian cancer, NSG mice were injected i.p. with 5 × 10^6^ SKOV3 fLuc+ cells. Twenty days after peritoneal inoculation, mice bearing well-established SKOV3 tumors were divided into groups and treated. Mice were sacrificed and necropsied when the mice became distressed and moribund. To monitor the extent of tumor progression, the mice were imaged weekly or biweekly and body weights of the mice were measured. In all models, 4–5 mice were randomized per group prior to treatment.

### Bioluminescence imaging

Tumor growth was also monitored by Bioluminescent imaging (BLI). BLI was done using Xenogen IVIS imaging system and the photons emitted from fLuc-expressing cells within the animal body were quantified using Living Image software (Xenogen). Briefly, mice bearing SKOV3 fLuc+ tumor cells were injected intraperitoneally with D-luciferin (150 mg/kg stock, 100 μL of D-luciferin per 10 grams of mouse body weight) suspended in PBS and imaged under isoflurane anesthesia after 5∼10 minutes. A pseudocolor image representing light intensity (blue, least intense; red, most intense) was generated using Living Image. BLI findings were confirmed at necropsy.

### Statistical analysis

The data are reported as means and SD. Statistical analysis was performed by the use of 2-way repeated-measures ANOVA for the tumor burden (tumor volume, photon counts). Student *t* test was used to evaluate differences in absolute numbers of transferred T cells, cytokine secretion, and specific cytolysis. GraphPad Prism 5.0 (GraphPad Software) was used for the statistical calculations. *P* < *0.05* was considered significant.

## SUPPLEMENTARY FIGURES


