# Supplementary material for: A fully human chimeric antigen receptor with potent activity against cancer cells but reduced risk for off-tumor toxicity
Source: Oncotarget. 2015 Jun 19;6(25):21533–46. doi: 10.18632/oncotarget.4071 (PMC4673284; doi:10.18632/oncotarget.4071)
Supplement: Supplementary file 1 [file oncotarget-06-21533-s001.pdf]

SUPPLEMENTARY FIGURES

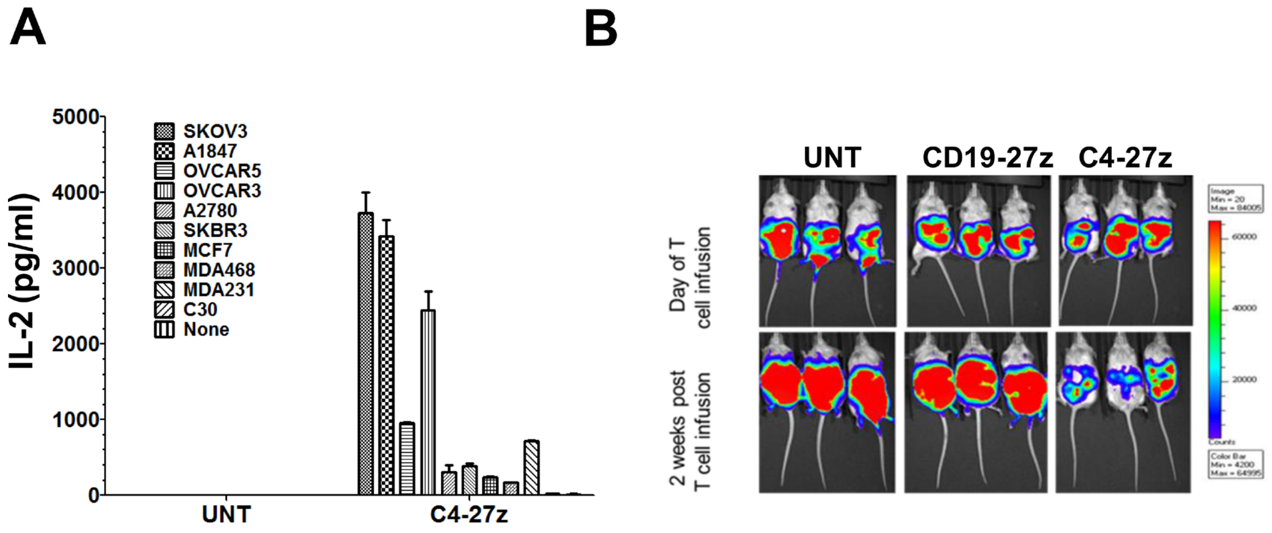

Supplementary Figure 1: A. C4-27z CAR but not UNT T cells (10^5 cells/well) secret IL-2 following overnight incubation with ovarian and breast cancer cell lines (105 cells/well) expressing different levels of surface αFR. Mean IL-2 concentration ± SEM (pg/ml) from triplicate cultures is shown. **B.** NSG mice received i.p. injection of 3×10^6 SKOV3 fLuc tumor cells and were randomized into 3 groups of 5 mice each before beginning therapy with UNT T cells or T cells expressing C4-27z or CD19-27z CAR via i.v. infusion on day 21 and 25 after tumor inoculation. Bioluminescence images show fLuc+ SKOV3 tumors in NSG mice immediately prior to and two weeks after last injection cell dose.

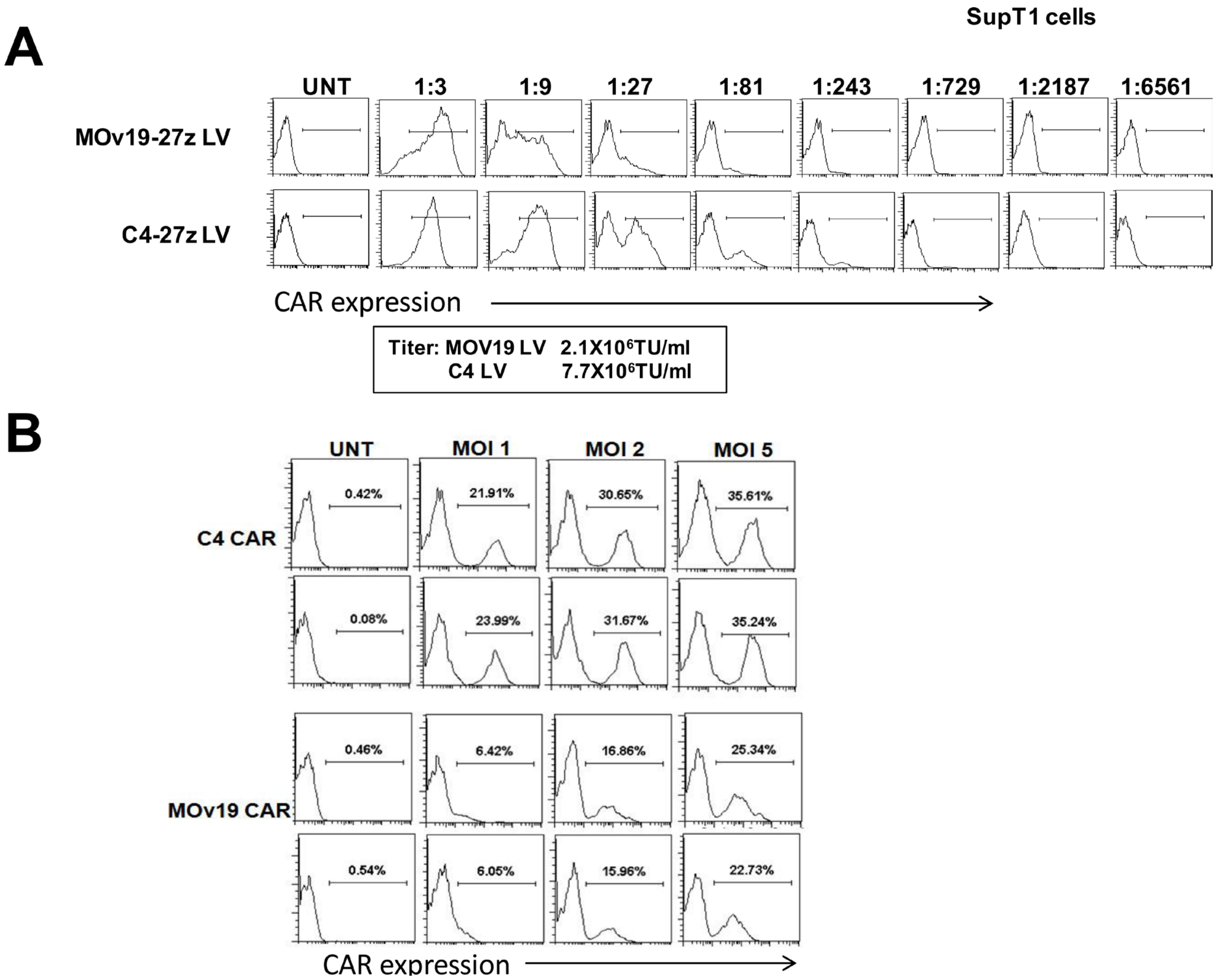

Supplementary Figure 2: Fully human C4 CAR can be easily expressed and detected on T cell surface. A. Lentiviral titers (transduction units, TU) were determined using SupT1 cells based on 3-fold serial dilution of concentrated virus from 1:3 to a final dilution of 1:6,561. Compared to the titer of MOv19 CAR encoding lentivirus, C4 CAR-encoding lentivirus has a higher titer when following the same production and concentration protocols in parallel. **B.** Primary human T cells were infected with C4 CAR or MOv19 CAR encoding lentivirus at a multiplicity of infection (MOI) of 1, 2 or 5. These data represent one of at least three independent experiments.

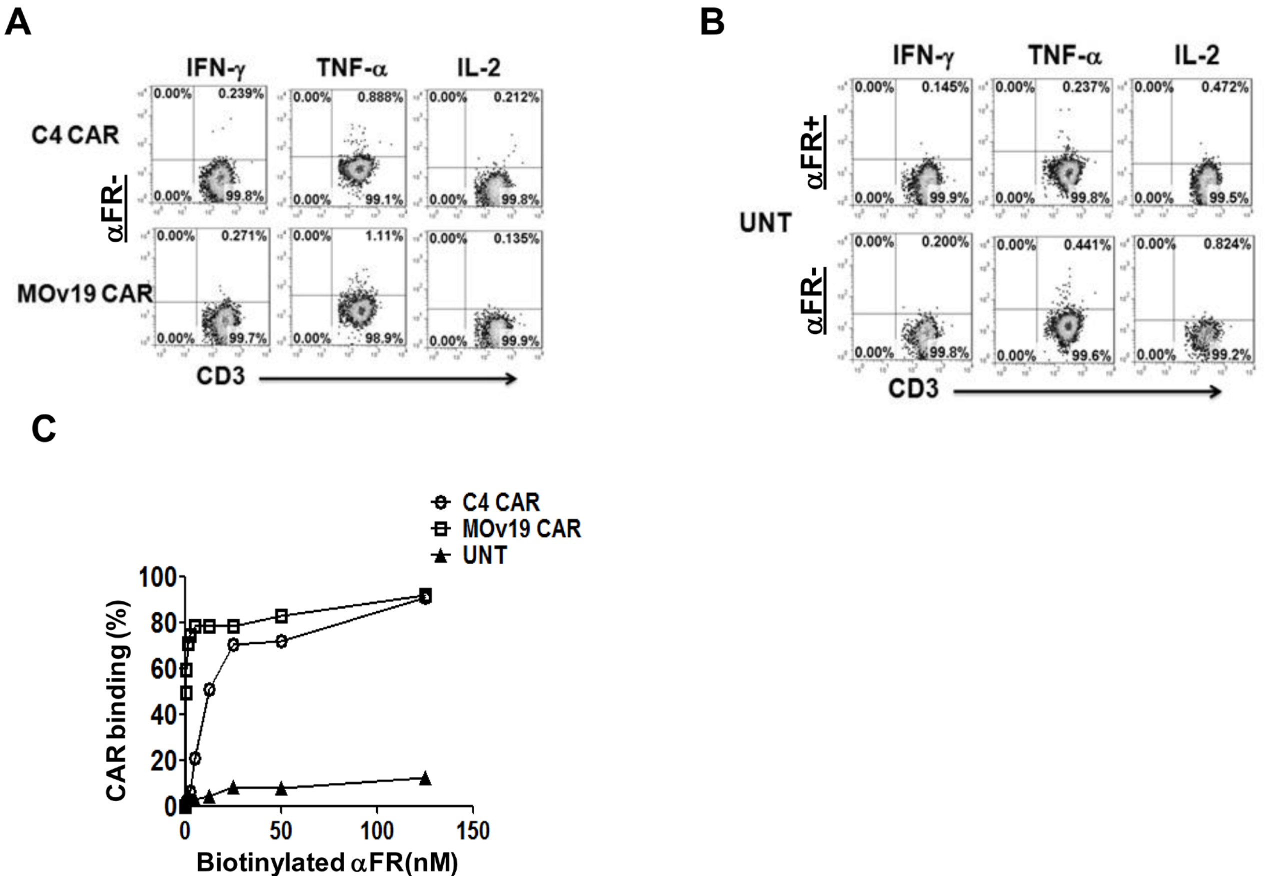

Supplementary Figure 3: A. C4 or MOv19 CAR T cells were stimulated with αFR- C30 cells, and **B.** untransduced (UNT) T cells were stimulated with aFR+ SKOV3 cells for 5-hour in the presence of Golgi inhibitor and analyzed by flow cytometry for intracellular IFN-γ, TNF-α and IL-2. **C.** Titration analysis on the binding of biotinylated αFR protein to αFR CAR T cells. Activated T cells were transduced with lentiviral vector expressing MOv19-27z or C4-27z-CAR and analyzed for CAR expression on day 14. One hundred thousand UNT or CAR T cells were stained with 0.2, 0.5, 1, 2, 5, 10, 20, 50 or 120 nM/sample of biotinylated αFR. T cells were then washed and stained with phycoerythrin (PE)-conjugated streptavidin (SA). T cells were analyzed using flow cytometer and the data analyzed with FlowJo software. Result of a representative experiment from three independent experiments is presented.

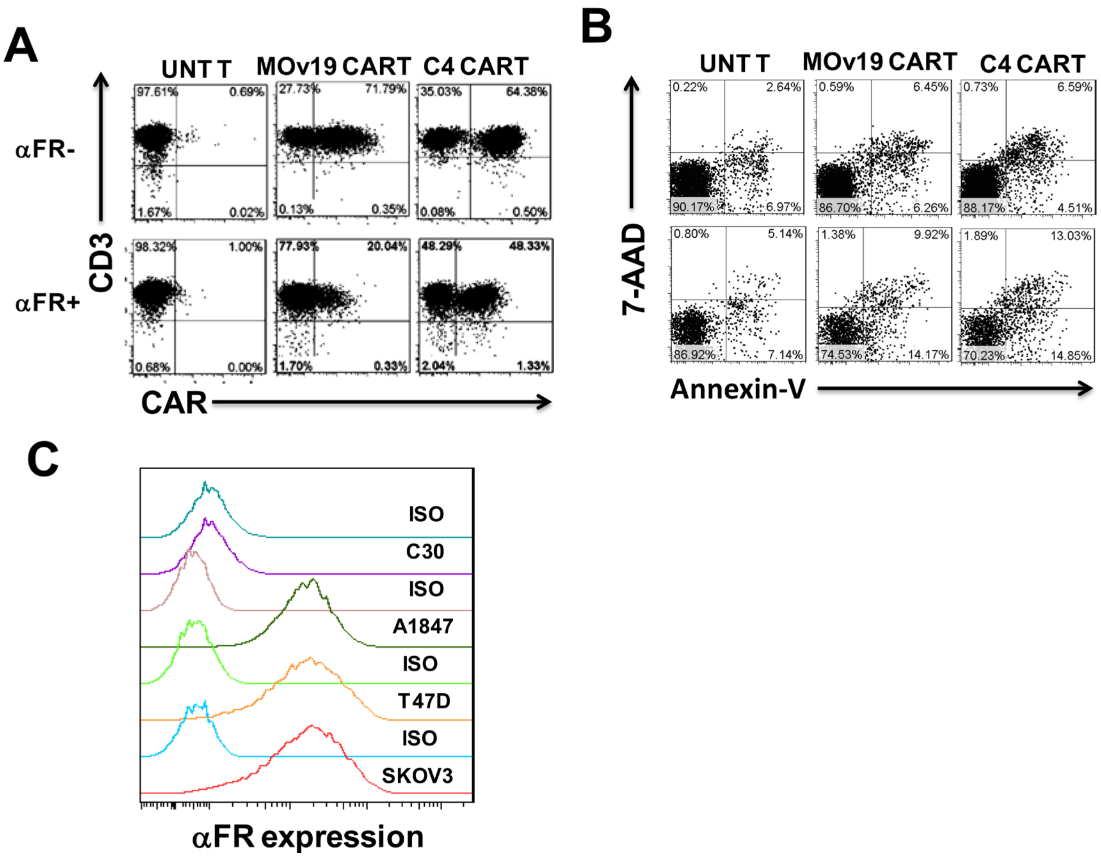

Supplementary Figure 4: A. Flow cytometry analysis of CAR expression changes after 4 h coculture with αFR+ or αFR- tumor cells. **B.** MOv19 and C4 CAR T cells cocultured with αFR+ or αFR- tumor cells for 4 h and then stained with annexin V and 7-AAD. The apoptotic cells are indicated as the percentage of gated cells. **C.** αFR expression on SKOV3, A1847 and T47D tumor cell lines; C30 cell line was used as a negative control.

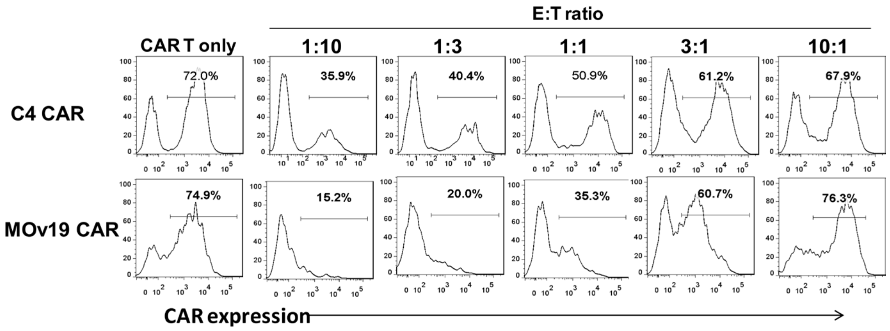

Supplementary Figure 5: Flow cytometry analysis of CAR expression changes after overnight co-culture with αFR+ tumor cells (at 1:10, 1:3, 1:1, 3:1 and 10:1 ratios).
